# Variant patterns and influence of inter-regional travel during the SARS-CoV-2 expansion in South Africa

**DOI:** 10.1371/journal.pone.0329621

**Published:** 2025-11-06

**Authors:** Weiyu Luo, Xin Wu, Ruohan Li, Meagan Fitzpatrick, Man Charurat, Natalia Blanco, Kristen A. Stafford, Vivek Naranbhai, Alash’le Abimiku, Anna Winters, INFORM Africa Research Study Group for D-SI Africa Consortium, Chenfeng Xiong

**Affiliations:** 1 Department of Civil and Environmental Engineering, College of Engineering, Villanova University, Philadelphia, Pennsylvania, United States of America; 2 School of Medicine, University of Maryland Baltimore, Baltimore, Maryland, United States of America; 3 CAPRISA - Centre for AIDS Programme of Research in South Africa, Congella, KwaZulu-Natal, South Africa; 4 School of Translational Medicine, Department of Medicine, Monash University, Melbourne, Australia,; 5 Institute of Human Virology Nigeria, Abuja, Nigeria; 6 Akros Research, Kabulonga, Lusaka, Zambia; Politecnico di Milano, ITALY

## Abstract

We evaluated the dynamic impacts of three types of human mobilities—provincial inflows, cross-district flows, and within-district flows—on daily reported COVID-19 cases for 2020. Using a structural equation modeling approach, we conducted regressions on dynamic panel datasets. Our findings indicate that these three types of mobility influenced daily new COVID-19 case numbers in distinct and sometimes overlapping ways during the early stages of the epidemic. Within-district flows played a particularly significant role in increasing cases during the spreading stage. During the epidemic stage, we observed a sustained but gradually declining impact of within-district mobility on daily new cases, potentially highlighting the effectiveness of non-pharmaceutical interventions (NPIs). In addition, signs of social distancing fatigue were evident. Our model further shows that the first and most stringent lockdown policy significantly curtailed human mobility, whereas the second, less restrictive lockdown had negligible impact on human mobility.

## 1. Introduction

We have grown accustomed to connecting globally through an extensive, high-frequency, fast, and safe transportation system. However, while this system has dramatically enhanced human mobility, it has also facilitated the spread of infectious diseases. Beginning from December 2019, when Coronavirus Disease 2019 (COVID-19) was first identified, the COVID-19 pandemic, characterized by its rapid spread, has impacted a staggering 768,560,727 individuals worldwide and tragically resulted in 6,952,522 fatalities as of July 26^th^, 2023 [1]. This global health crisis has deteriorated into one of the most severe challenges faced by the worldwide community in recent decades. Lockdown policy was widely used by governments to restrict travel and contain the spread of the virus. The restriction has been proven to be effective in slowing down the spread of the virus [2,3]. The COVID-19 pandemic has compelled a re-evaluation of the relationship between transportation and economic activity, work and lifestyle, and even interpersonal communication [4–6]. In this context, studying human mobility patterns is crucial in combating the epidemic.

According to data reported to the World Health Organization (WHO), in South Africa, spanning from January 3^rd^, 2020, to July 26^th^, 2023, a total of 4,072,533 confirmed cases of COVID-19 have been documented, resulting in 102,595 deaths [7]. The first confirmed case of COVID-19 in South Africa emerged in KwaZulu-Natal province on March 5^th^, 2020. Starting on March 15th, the South African government swiftly responded to the COVID-19 pandemic by declaring a State of National Disaster, closing ports of entry, and issuing travel advisories. They also published guidelines to reduce social interaction and identified essential services that would remain operational during the lockdown period. On March 27th, 2020, the government implemented the strictest lockdown measure (Alert Level 5) to restrict travel. This level of alert is described as “Drastic measures to contain the spread of the virus and save lives” [8]. As the pandemic escalated, the government increased testing capacity, secured critical protective equipment, extended the lockdown through April, postponed exams, and addressed the needs of vulnerable populations. By May 2020, the government began gradually relaxing lockdown conditions through a risk-adjusted strategy, introduced economic and social relief measures, and recommended the public use cloth masks [9]. Mask-wearing became prevalent from May to August 2020 as COVID-19 cases surged [10], with the total number of cases rising exponentially to over 500,000 by August 1st, 2020 [7]. Until February 17^th^, 2021, South Africa administered its first COVID-19 vaccine [11].

In the absence of a vaccine in year 2020, non-pharmaceutical interventions (NPIs) became essential to restrict the people’s physical movements and gathering to control the rising cases of COVID-19, with substantial potential to reduce infections among individuals [12]. Previous studies on Europe [13], China [2,3,14], India [4], Japan [15] and the U.S. [16] demonstrate the critical role of NPIs, particularly during the initial stages of an epidemic. In South Africa, model results indicate that an early or rapid relaxation of NPIs would be at high risk, likely precipitating a substantial infection wave, and the vaccination alone is insufficient to contain the outbreak [17]. NPIs remain particularly critical for vulnerable populations, as a study in South Africa highlights the need for older adults and individuals with chronic illnesses to adopt additional preventive measures, such as telework and avoiding social gatherings or public spaces, to minimize transmission risk [18]. Although the national lockdown interventions in South Africa significantly reduced the case numbers, supercritical growth was still observed post-lockdown [19], highlighting the need to analyze the change in human mobility patterns under lockdown policies during the pandemics.

To explore human mobility and travel behavior in general, prior studies have utilized diverse data sources, including Call Detail Record (CDR) data [20], mobile device Location-Based Service (LBS) data [21,22], social media data [23], GPS locations [24] and survey data [25]. During the COVID-19 pandemic, Facebook mobility data [26,27], Google mobility data [15], Safegraph [28], Twitter data [23], survey datasets [29,30] and LBS data [22,31] were measured as proxies of human mobility to understand the pattern change. In the context of South Africa, for example, survey data revealed that respondents in South Africa significantly reduced travel for leisure activities, such as purchasing nonessential goods, visiting relatives, or socializing with friends [29]. Potgieter et al. (2021) compared LBS data, Facebook mobility data and other data as sources to calculate spatial weight matrix which measures the dependency between spatial units in South Africa [27]. However, this study did not model the relationship between viral transmission and human mobility. Manda et al. (2021) uses spatial regression to analyze COVID-19 prevalence across African countries, considering socio-economic factors and neighboring effects [32], but they did not model the temporal dynamics. In other countries, prior studies have processed the LBS data and built models to explore the effects of human mobility on COVID-19 infections [3,22,31,33,34], vice versa [21,35] and the effects of lockdown policy on human mobility [36] in China and U.S. In this paper, we utilize LBS data for the study of South Africa because they are less costly, have a higher penetration rate, wider date coverage and better temporal resolution compared to other data sources [37].

Several research gaps still exist and could be filled with this study. First, although South African Government has implemented a nationwide lockdown policy, human mobility can still be affected by the way local governments implement the policy, which in turn affects the path of virus transmission. In order to understand how human mobility changes within or between different administrative regions, we extracted Origin-Destination (O-D) information for these regions from the LBS data and use it to derive human mobility metrics. Estimating human mobility flow from O-D data is a widely recognized approach. For instance, Kang et al. (2020) published COVID-19 U.S. flow data aggregated at Census Block Group (CBG) level [38]. They proposed *visitor flow metric* as proxy to measure the human mobility between geographic units. To the best of the authors’ knowledge, this study is the first to derive human mobility metrics with O-D information from Location-Based Services (LBS) data for South Africa. No prior studies categorize human mobility based on whether it crosses specific administrative boundaries or assesses its impact on virus transmission. Second, while some studies [22] have examined the dynamic effects of human mobility on COVID-19 cases, none consider lockdown policies as a dynamic factor in human mobility. Third, although previous literature has explored infection rates [39] and population mobility [27], there is no study quantifying the influences of human mobility on COVID-19 cases in South Africa. To fill these important gaps, our experiment fitted a structural equation model (SEM) on dynamic datasets of South Africa to capture the dynamic effects. SEM is a statistical technique for modeling complex relationships among variables, testing hypotheses about direct and indirect effects [40]. In this study, we aim to answer these research questions below:

- How do different types of human mobility affect the virus transmission?
- How does the impact of different types of human mobility on virus transmission vary across different pandemic stages?
- How does the impact of lockdown policies on human mobility evolve over time?

The remainder of this paper is organized as follows:

- Section 2 describes the endogenous and exogenous variables.
- Section 3 is dedicated to the modeling methodology, with a focus on formulating the dynamic SEM.
- Section 4 presents the results with detailed explanation on different stages of the epidemic.
- Section 5 concludes the key findings, contributions, limitations and future studies.

## 2. Data description

South Africa is divided into nine provinces, which are further subdivided into 52 districts, as illustrated in Fig 2. The boundaries of these provinces and districts are determined by the Municipal Demarcation Board, which adheres to political, administrative, and statistical criteria to ensure effective governance and service delivery, as noted by the South African National Census framework [41,42]. According to Statistics South Africa’s Census 2022 [43], the average population per province is approximately 6.9 million, with Gauteng being the most populous at 15 million and the Northern Cape the least at 1.3 million, while the median provincial population is around 6.6 million (e.g., Limpopo at 6.6 million). At the district level, the average population is roughly 1.1 million, with a median of about 811,000 [42]. The discrepancy reflects significant variation due to urban-rural divides (e.g., metropolitan districts like Johannesburg versus rural districts in the Northern Cape).

**Fig 1:**
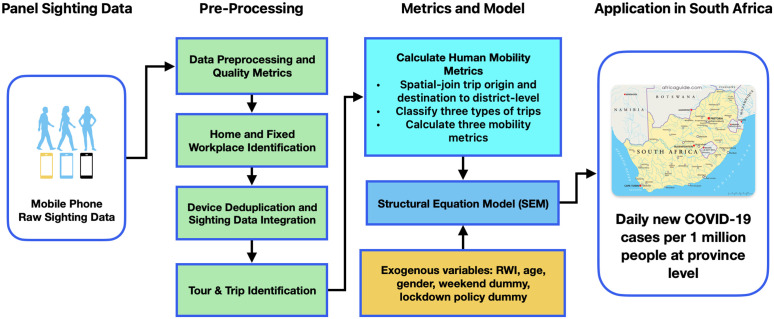
A big-data-driven analytical framework of human mobility and pandemics.

In this section, we introduce three key human mobility metrics—Type 1, 2 & 3 mobility metrics—which form the backbone of our data analysis framework. Each metric is carefully designed to capture unique aspects of the dataset, enabling a multidimensional understanding that is essential for subsequent analysis. The Type 1 mobility metric measures human mobility inflows from other provinces into a specific province, capturing the external impact of predominantly long-distance trips. The Type 2 mobility metric quantifies cross-district mobility within a specific province, assessing internal impacts related to mid-distance movement. Lastly, the Type 3 mobility metric measures mobility within district boundaries in a province, focusing on internal impacts associated with short-distance travel.

The human mobility metrics are generated by applying a big-data-driven analytical framework. Starting from raw location sighting data sourced from mobile devices, we developed various quality metrics to confirm the data frequency, stability, and data consistency. We filtered raw data only from pre-defined Regular Active Users (RAUs) to identify subsequent mobilities. An RAU must be observed in no less than eight different unique hours on at least one day in a month. An average of 284,684 RAUs were selected in the 2020 dataset for all months, which is approximately 0.48% of the total population of 58.8 million. Home, fixed workplaces, tours, and trips were then identified based on a series of validated algorithms. Home and workplaces are inferred by a validated rule-based algorithm that counts daytime and nighttime sightings to make inferences. Tours and trips are inferred through a recursive rule-based algorithm that utilizes distance, time and speed between consecutive sighting data. The algorithms are applied on the sightings from all RAUs, and all inferred trips are included in the modeling. Trips are the finest unit to derive the mobility metrics. The process is illustrated in Fig 1. The parameters for home, work, tour and trip identification in this study and more detailed information can be found from this report https://nhts.ornl.gov/od/assets/doc; https://scholar.google.com/citations?view_op=view_citation&hl=en&user=7u8XzEMAAAAJ&sortby=pubdate&citation_for_view=7u8XzEMAAAAJ:roLk4NBRz8UC; https://github.com/citation-style-language/schema/raw/master/csl-citation.json [44]. The 2020 human mobility metrics are generated at district-level in South Africa. The research approach adopted in the study, including data sources, data processing, and modeling methodologies, was reviewed and approved by the Institutional Review Boards at Villanova University and the University of Maryland Baltimore, and the National Health Research Ethics Committee of Nigeria (NHREC). Due to a non-disclosure agreement with the data provider, the specific source of the LBS data cannot be named; however, the dataset is collected from anonymized mobile devices over year 2020 across South Africa region.

### 2.1 Endogenous variables

The COVID-19 case data were retrieved from Coronavirus COVID-19 (2019-nCoV) Data Repository for South Africa [45,46] of year 2020. Cumulative case counts were converted to daily new cases using first-order differencing. Provincial daily new COVID-19 cases were then standardized by dividing by the provincial population, yielding cases per 1 million people.

**Fig 2:**
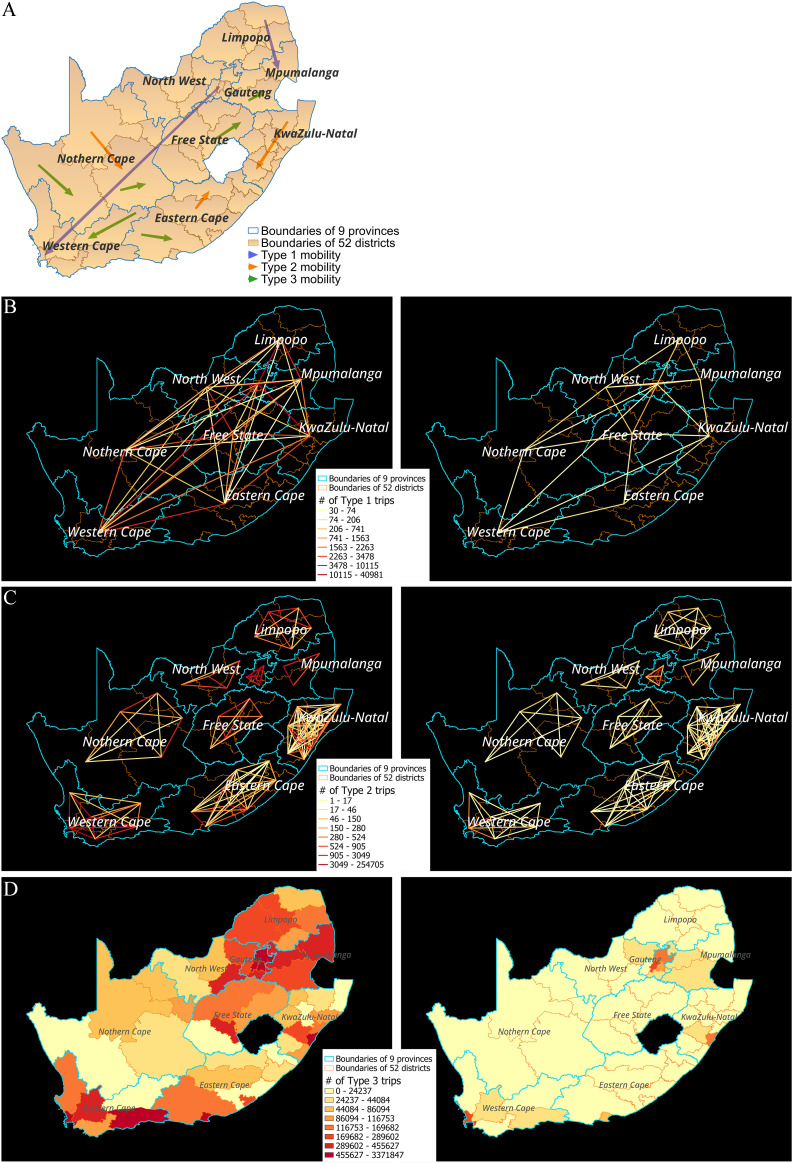
(a) illustration of 3 types of human mobility flow. (b) Number of Type 1 trips comparison. (c) Number of Type 2 trips comparison. (d) Number of Type 3 trips comparison. The comparison is between pre-pandemic period (1/1/2020–1/31/2020) & post-pandemic period (3/27/2020–4/26/2020).

South Africa is divided into nine provinces, which are further subdivided into 52 districts, as illustrated in Fig 2. The blue and brown boundaries represent provinces and districts, respectively. Fig 2 also illustrates three types of trips incorporated into the model: province-inflow trips, cross-district trips, and within-district trips, represented by blue, orange, and green arrows. Using billions of location-based service data, we developed various mobility informatics in the data-driven analytical framework to better understand human mobility patterns. Three types of mobility metrics were defined as below and illustrated in Fig 2(a). Fig 2(b)–(d) display the three types of identified trips during the month before (1/1/2020–1/31/2020) and after (3/27/2020–4/26/2020) the outbreak.

(1) Type 1 mobility metric: Total number of identified trips from other provinces each day divided by province population (per million people).
(2) Type 2 mobility metric: Total number of identified trips of which origin and destination (O-D) were in different districts but within the same province on each day divided by province population (per million people).
(3) Type 3 mobility metric: Total number of identified trips of which origin and destination (O-D) were both in the same district on each day divided by province population (per million people).

Individual-level trips were aggregated by the districts of their origins and destinations, and then by provincial destinations to derive grouped provincial metrics. This study employs the three types of mobility metrics mentioned above to model the dynamic effects between daily new COVID-19 cases and human mobility. A summary of these variables is presented in Table 1. To be noted, by comparing the minimum/maximum percentage of the three metrics, the percentages are 0.03%, 0.02% and 0.91% for Type 1, 2 and 3 metric, respectively. The percentage of Type 3 is much larger than Type 1 and Type 2, which implies that even under extreme conditions, there is a minimum level of demand for short-distance travel to keep alive.

**Table 1:** Descriptive statistics of endogenous and exogenous variables.

Variables	Description		Mean	Std	Min	Max
**Endogenous variables**						
Daily new cases	Daily new COVID-19 cases divided by province population (per million people)		49.16	82.77	0.00	614.79
Type 1 mobility metrics	# of identified trips from other provinces on each day divided by province population (per million people)		127.54	128.58	0.29	833.80
Type 2 mobility metrics	# of identified trips of which origin and destination were in different districts but within the province on each day divided by province population (per million people)		267.00	472.04	0.75	3169.14
Type 3 mobility metrics	# of identified trips of which origin and destination were both in the same district on each day divided by province population (per million people)		6016.74	5615.64	337.78	36711.51
**Exogenous variables**						
RWI	Averaged Relative Wealth Index (RWI) of a province in South Africa		0.17	0.24	−0.05	0.67
Age_0_14	Percentage of population below age 14		0.29	0.03	0.23	0.33
Age_65+	Percentage of population above age 65		0.06	0.01	0.05	0.08
Male_pct	Percentage of male population		0.49	0.01	0.47	0.52
Weekend	A dummy variable = 1 if the date is weekend.		0.28	0.45	0.00	1.00
Lockdown level		Alert level of the lockdown policy. Eligible values include {0, 1, 2, 3, 4, 5}. This variable is used to group samples to estimate the random effects in each windowed panel data.				

**Fig 3:**
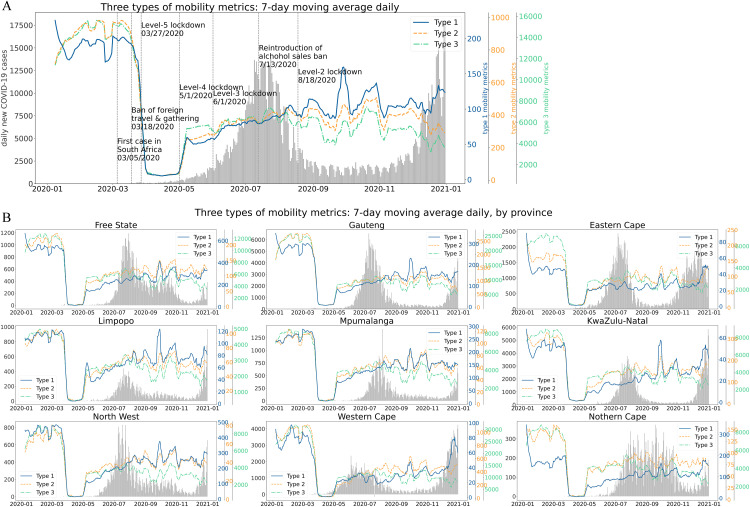
(a) Varying pattern of the three types of mobility metrics of South Africa (weighted average by province population) and daily reported new COVID-19 cases from March 5th, 2020, to December 31st, 2020. (b) Separated by provinces of South Africa.

Fig 3(a) visualizes the daily varying pattern of the three types of mobility metrics in South Africa, with daily new COVID-19 cases shown as a grey background bar. All three metrics exhibit sharp declines between the travel ban announcement (March 18^th^, 2020) to April 1^st^, 2020. Following the relaxation of lockdown to level 4 (May 1^st^, 2020), all three metrics surged, with mobility gradually recovering until the reintroduction of the alcohol sales ban (July 13^th^, 2020). During this period, daily new COVID-19 cases increased exponentially, peaking on July 9^th^, 2020. After July 13^th^, the patterns of the three metrics began to diverge, with Type 3 metric showing a more pronounced decline compared to the other two types. One explanation is that people consciously limited daily short-distance travels to avoid gathering, in response to the recent surge of new cases and government policies. Fig 3(b) shows the heterogeneity of the evolution of the three mobility metrics in each province during the pandemic. The provinces show different patterns at different stages of the 2020.

To validate the data for modeling, we calculated the trips per person per day of January 2020 data and compared it with the National Household Travel Survey (NHTS) of the year 2013 [47]. The average trips per person per day in January 2020 is 2.26, aligning closely with the 2013 NHTS result of 2.51 trips per person per day in South Africa. This consistency suggests that the framework generated reasonable trip rosters.

### 2.2 Exogenous variables

Besides the four variables mentioned above, multiple other factors are introduced as covariates into our model. The Relative Wealth Index (RWI) [48] serves as an indicator of the general economic conditions across South African provinces. RWI was developed to micro-estimate the relative wealth and poverty levels in low- and middle-income countries at a 2.4 km resolution. In this research, the RWI value of each province is generated through the following steps. Step 1, all 2.4-km-resolution RWI points are spatially joined in all districts and grouped by districts. The mean RWI of all districts is then calculated. Step 2, The districts are then grouped by their corresponding provinces, with the RWI for each province determined as the population-weighted average of the RWI values for the districts within that province. Sociodemographic data [49], including age distribution and sex ratio, are sourced from the South Africa Community Survey. Dummy variables of the first reported case and the travel ban policy are from publicly available information [50]. The lockdown level variable is defined by the South African government, with level 5 being the most restrictive and level 0 indicating no lockdown. Detailed description of these alert levels can be found here [8].

## 3. Modeling

### 3.1 Ethics statement

This study was approved by the Villanova University Institutional Review Board (IRB #: IRB-FY2023–145). All procedures adhered to ethical standards for the protection of human subjects.

### 3.2 Dynamic panel data

The entire dataset in this study is from January 1^st^, 2020, to December 31^st^, 2020. For each day T, the SEM model was fitted in a dynamic panel data over the period of [T, T+B), where B=120 days. This 120-day dynamic panel starts from $T=\text{March }{\text{5}}^{th}\text{, 2020}$, slides by day and ends until the last panel exhausts the entire dataset, i.e., $T+B=\text{January }{\text{1}}^{st}\text{, 2021}$. In total, there are 181 dynamic panel datasets. The reasons to set B=120 are two-fold. Firstly, we want to include lockdown-level as covariates to enhance our sub-models for the human mobility metrics, as it is believed that people’s travel behaviors were significantly affected by the lockdown policies during the pandemic [3,35,36]. Therefore, the lockdown-level variables cannot be constant in any of the dynamic panel datasets. In 2020, the longest duration between two different lockdown levels was 101 days, spanning from lockdown level 1 on September 21st to December 31st. Therefore, a 120-day (or 4-month) period was selected to cover this interval to ensure the variables are not constant. Secondly, we wanted to expand the sample size, as modeling at the provincial level resulted in a sample size of only nine for each day of T.

### 3.3 Formulation of dynamic structural equation model (SEM)

To capture the time-varying direct effects on the daily new cases from Type 1, Type 2 and Type 3 metrics, as well as the indirect effects of socio-demographic and policy variables, we employed an SEM with dynamic panel and time-varying coefficients. SEM allows variables to be both predictor and response variables, thus quantifying indirect effects. These effects cannot be recognized by any other single-equation model [40]. This SEM is composed of four linear mixed-effect sub-models to capture both fixed and random effects of the variables. The structure of the model is realized by using an R package called “piecewiseSEM” [40]. For each dynamic panel dataset over the period of [T, T+B), the formulation of the model is described in Equation (1):


$$\text{In}\;y_{i\text{,}t}=c_{T}+\alpha_{T}\;\text{In}\;y_{i\text{,}t-\text{1}}+\sum_{N}\beta_{n\text{,}T}\;\text{In}\;X_{n\text{,}i\text{,}t-p_{n}}+\sum_{M}\gamma_{m\text{,T}}Z_{m\text{,}i}+\delta_{T}W_{i\text{,}t}+u_{i,T}+e_{i\text{,}t,T}$$ (1)

N={1(Type 1 metrics),2(Type 2 metrics),3(Type 3 metrics)}

M={1(RWI),2(Age 65+),3(Male pct)}

In equation (1):

- $y_{i,t}$ represents the dependent variable of province i at date t.
- $c_{T}$ is the constant term serving as the intercept of the model for each dynamic panel data on the period of [T, T+B).
- $X_{n,i,t-p_{n}}$ is the variable of type n mobility metrics from set N of province i.
- $\beta_{n,T}$ is the corresponding coefficient for the variable and the dynamic panel data on the period of [T, T+B).
- $Z_{m,i}$ is $m^{th}$ time-invariant variable from set M of province i and $\gamma_{m,T}$ is the corresponding coefficient.
- $W_{i,t}$ stands for weekend dummy variable and $\delta_{T}$ is the corresponding coefficient.
- $u_{i,T}$ denotes the random effect term, which is independent of all $y_{i,t}$, $X_{n,i,t-p_{n}}$ and $Z_{m,i}$ but common to each province i over the period of [T, T+B).

For the variables $X_{n,i,t-p_{n}}$ that are used as predictor variables in equation (1), they are also used as response variables in the sub-models as shown in equations (2)–(4):


$$\begin{array}{c} \text{In}\;X_{\text{1,}i\text{,}t-p_{\text{1}}}=c_{T}^{\prime }+\alpha_{T}^{\prime }{\text{SMA}(\text{In}\;y_{i\text{,}t-p_{\text{1}}})}_{k}+\beta_{T}^{\prime }\;\text{In}\;X_{\text{1,}i\text{,}t-p_{\text{1}}-\text{1}}\\ +\sum_{M}\gamma_{m\text{,}T}^{\prime }Z_{m\text{,}i}+\delta_{T}^{\prime }W_{i\text{,}t-p_{\text{1}}}+\sum_{L}V_{l,T}^{\prime }+\sum_{L}u_{i\text{,}l,T}^{\prime }+e_{i\text{,}t-p_{\text{1}},T}^{\prime } \end{array}$$ (2)

$$\begin{array}{c} \text{In}\;X_{\text{2,}i\text{,}t-p_{\text{2}}}=c_{T}^{\prime \prime }+\alpha_{T}^{\prime \prime }{\text{SMA}(\text{In}\;y_{i\text{,}t-p_{\text{2}}})}_{k}+\beta_{T}^{\prime \prime }\text{In}\;X_{\text{2,}i\text{,}t-p_{\text{2}}-\text{1}}\\ +\sum_{M}\gamma_{m\text{,}T}^{\prime \prime }Z_{m\text{,}i}+\delta_{T}^{\prime \prime }W_{i\text{,}t-p_{\text{2}}}+\sum_{L}V_{l,T}^{\prime \prime }+\sum_{L}u_{i\text{,}l,T}^{\prime \prime }+e_{i\text{,}t-p_{\text{2}},T}^{\prime \prime } \end{array}$$ (3)

$$\text{In}\;X_{\text{3,}i\text{,}t-p_{\text{3}}}=c_{T}^{'''}+\alpha_{T}^{'''}{\text{SMA}(\text{In}\;y_{i\text{,}t-p_{\text{3}}})}_{k}+\beta_{T}^{'''}\text{In}\;X_{\text{3,}i\text{,}t-p_{\text{3}}-\text{1}}+\sum_{M}\gamma_{m\text{,}T}^{'''}Z_{m\text{,}i}+\delta_{T}^{'''}W_{i\text{,}t-p_{\text{3}}}+\sum_{L}V_{l,T}^{'''}+\sum_{L}u_{i\text{,}l,T}^{'''}+e_{i\text{,}t-p_{\text{3}},T}^{'''}$$ (4)

$${SMA(x_{t})}_{k}=\frac{\left(x_{t-\text{k}}+\ldots +x_{t-1}\right)}{k}$$ (5)

L={5(Most\ strict\ lockdown\ level),4, 3, 2, 1(Least\ strict\ lockdown\ level)}

In equation (2): 

- $V_{l,T}^{\prime }$ represents the random effect term of lockdown level l from set L.
- $u_{i,l,T}^{\prime }$ denotes the random effect term of province i at lockdown level l over the period of [T, T+B).
- “SMA” denotes “Simple Moving Average” function as shown in equation (5). This function is applied in the time-series of each province for k steps. We set k = 7 here to smooth the weekly pattern.

The set L consists of the eligible lockdown levels during the period of [T, T+B). Natural logarithmic transformation is employed for all endogenous variables to make the data more normally distributed.

$p_{1},p_{2},p_{3}$represent the time lags of 3 types of metrics in Equation (1), describing the lagged effects on daily new cases. We calculated the correlations between daily trip counts of each type from 1-day lag to 30-day lag and daily new cases. These lag steps, $p_{1},p_{2},p_{3}$, are determined by selecting the largest correlation among the 30 choices [51,52]. They are determined as below.


$$p_{\text{1}}=\text{3, }p_{\text{2}}=\text{16, }p_{\text{3}}=\text{22}$$

The variance inflation factor (VIF) was employed to test the multicollinearity for each sub-model of the SEM. The results show that VIFs of all variables in all sub-models are lower than 5.0, indicating a low correlation of each variable with other variables among all sub-models. Details of the VIF values can be found in the Supporting Information S1–S4 Tables.

## 4. Results

### 4.1 Goodness-of-fit

Table 2 presents the conditional R-squared statistics for the sub-models of daily new COVID-19 cases, Type 1 metrics, Type 2 metrics, and Type 3 metrics. Table 2 shows that of the 181 dynamic panel datasets, the means of conditional R-squared are all above 0.86 among all four sub-models. Among all models which were applied on 181 panel datasets, the lowest R-squared value is 0.76. It indicates that the SEMs fit well among all dynamic panel datasets. A Durbin-Watson test was performed on the model residual of all the 120-day time-series data of every province. The results show that 99% of the time series have a Durbin-Watson statistic between 1.0 and 3.0, and 75% fall between 1.5 and 2.5. This suggests that there is no significant autocorrelation in most of the model residuals, affirming the robustness of the R-square metrics.

**Table 2:** Statistics of sub-models’ R-squared on dynamic panel datasets.

Sub-model \ R-squared	Count	Mean	S.D.	Min	Max
Daily new cases	181	0.85	0.06	0.77	0.93
Type 1 metrics	181	0.93	0.02	0.88	0.95
Type 2 metrics	181	0.96	0.01	0.94	0.98
Type 3 metrics	181	0.89	0.06	0.81	0.97

### 4.2 Daily new cases v.s. Type 1, 2 and 3 metrics

**Fig 4:**
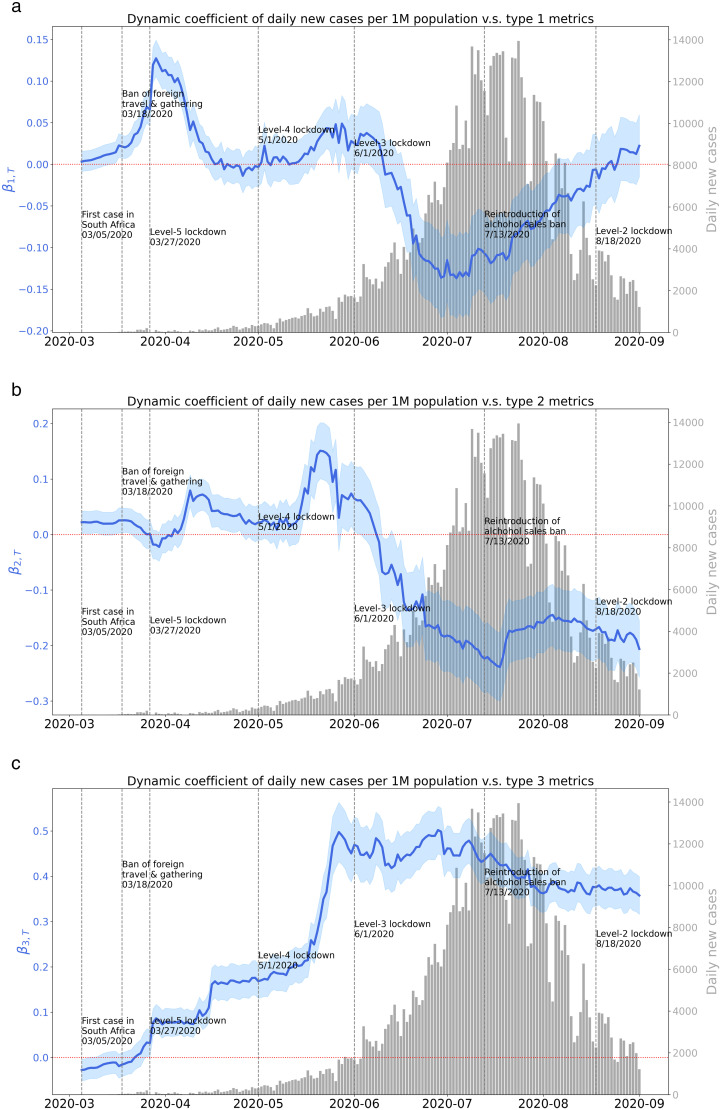
Dynamic coefficients between daily new COVID-19 cases per 1 million population and Type 1, 2, or 3 mobility metrics.

Fig 4 visualizes the daily variation of dynamic coefficients $\beta_{1,T},\;\beta_{2,T}\;and\;\beta_{3,T}$ in equation (1) in sub-figure (a), (b) and (c), respectively. The blue line represents the coefficients variation, the blue buffer represents the standard error of the coefficient. P-values for the coefficients are shown in S1–S3 Figs in the Supporting Information, where the red horizontal dashed line value is 0.05 for reference. The grey bar in the background denotes the daily new COVID-19 cases in South Africa. Black dotted vertical lines represent the dates of human-mobility-related events during the pandemic.

#### 4.2.1 Emergent stage (March 5^th^ to April 15^th^).

The variation of dynamic coefficient between daily new cases and Type 1, 2 or 3 mobility metrics is shown in Fig 4(a), (b) or (c). At the early stage of the pandemic, the P-value of coefficient of all three types of metrics fluctuate above 0.05, indicating insignificant impacts of three types of human mobility on daily new cases. Starting from March 22^nd^ to April 8^th^, the P-value of Type 1 metric sharply drops down and below 0.05, meanwhile the corresponding dynamic coefficient starts to rise from 0.04 on March 24^th^ to a peak at 0.13 on March 29^th^, then drops down to 0.06 on April 8^th^. This suggests that the daily new cases during this period can be associated with the provincial inflow trips of 3 days ago, with varying degrees of daily impact. Over the period of March 22^nd^ to April 8^th^, the average of the dynamic coefficients is 0.09. It indicates that if Type 1 mobility metrics increased by one-unit during the period, given other variables being equal, the daily new cases per 1M population would increase 0.09 on average.

Over the period of April 9^th^ to April 15^th^, the coefficient of Type 2 metric increases to 0.08 in average with the P-value dropping under 0.05. This period is immediately following the period of March 24^th^ to April 8^th^, showing a temporal alternation of influences of Type 1 metric and Type 2 metric. On March 28^th^, which is one-day after level 5 lockdown policy was issued, the dynamic coefficient of Type 3 metric starts to rise from 0.03 with the corresponding P-value sharply drops down and below 0.05. The dynamic coefficient of Type 3 metric remains positive from March 28^th^ until the last dynamic panel dataset beginning on September 1^st^ and the corresponding P-value remains below 0.05. This suggests that the daily new cases were significantly associated with within-district trips over this period. The corresponding dynamic coefficient, which measures the degree of impact, varies on a daily basis and this impact is observed significantly throughout almost the entire COVID-19 timeline of South Africa. Overall, the three mobility metrics show alternating or overlapping effects on daily new cases in different periods. The daily new cases were impacted by provincial inflow trips at first, then overlapped with the impact from within-district trips or alternated to cross-district trips. The date ranges when dynamic coefficients’ P-value of all mobility metrics was < 0.05 during emergent stage are shown in Table 3.

**Table 3:** Date range when dynamic coefficients’ P-value < 0.05 during emergent stage of the mobility metrics.

Type of metrics \ Date range	Mar 5^th^ – Mar 21st (17 days)	Mar 22^nd^ – Mar 27^th^ (6 days)	Mar 28^th^ – Apr 8^th^ (12 days)	Apr 9^th^ – Apr 15^th^ (7 days)
**Type 1**	No	Yes	Yes	No
**Type 2**	No	No	No	Yes
**Type 3**	No	No	Yes	Yes

#### 4.2.2 Spreading stage (April 16^th^ to June 1^st^).

As shown in Fig 4(c), a small jump can be observed on April 16^th^. The dynamic coefficient of Type 3 metric increases from 0.11 to 0.16, while the coefficients of Type 1 and Type 2 metrics decrease. One possible explanation is the diminishing impact of long-distance human mobility on the spread of the virus because of the lockdown policy and the gradual predominance of short-distance human mobility as a result of people’s daily needs. Due to this detected jump, we defined April 16^th^ as the start day of spreading stage. A more substantial spike is observed from May 16^th^ to May 27^th^, with the dynamic coefficient of Type 3 metric surging from 0.21 to 0.50, indicating that the impact of an additional unit of Type 3 metric on daily COVID-19 new cases increases from 0.21 to 0.50 during the period. Concurrently, the dynamic coefficient of Type 2 metric increases from 0.07 to a peak of 0.15 with the P-value is below 0.05. This suggests that Type 2 metric also exerts a growing influence during this spreading period. In contrast, the P-value of Type 1 metric is above 0.05 with positive dynamic coefficient values. It denotes that it still contributes a positive yet insignificant impact on daily new case number.

#### 4.2.3 Epidemic stage (June 2^nd^ to August 17^th^).

From May 28^th^ to July 13^th^, the dynamic coefficient of Type 3 metric fluctuates slightly between 0.42 and 0.50 with a mean of 0.46. It denotes that if Type 3 mobility metric increased by one-unit during the period, given other variables being equal, the daily new cases per 1M population would increase 0.46 in average. The coefficient gradually declined from 0.45 to 0.36 between July 14^th^ and August 16^th^, following the reintroduction of the alcohol sales ban policy. One possible explanation is that the new cases reduced following the new policy on July 13^th^. Additionally, the social-distancing and other NPIs as strategies to resist the virus became more widely accepted as part of their daily lives [10]. Consequently, a one-unit increase in Type 3 mobility metric had a reduced positive impact on the increase in daily COVID-19 new cases during the period.

The coefficient of Type 1 metric becomes negative over the period of June 22^nd^ to July 22^nd^, and the corresponding P-value falls below 0.05 during this period. The coefficient of Type 2 metric also becomes negative throughout June 24^th^ to September 1^st^ with the corresponding P-value falling below 0.05. One possible explanation is that people adjusted their routines by reducing short-distance travel to avoid gatherings, while increasing long-distance travel to distance themselves from areas with higher population density. These strategies may have diminished the metrics’ positive effects on daily new case numbers.

#### 4.2.4 Controlled stage (August 18^th^ to November 1^st^).

Compared with the previous stage, the dynamic coefficient of Type 3 metric drops to a lower range and becomes relatively stable between 0.36 and 0.39 from August 1^st^ to September 1^st^. It indicates that the dynamic impact of within-district trips on daily new cases reduced and became stable during the period. One possible explanation is that social distancing and other NPIs had become widely adopted as strategies to control the virus’s spread.

#### 4.2.5 Summary.

To summarize, the three mobility types had alternating or overlapping effects on daily new cases, with Type 1 mobility influential early on, Type 2 mobility gaining significance in the spreading stage, and Type 3 mobility having a sustained impact from early stage, intensifying in the spreading stage, peaking in the epidemic stage and weakening in the controlled stage. To be noted, the absolute number of Type 3 mobility metric is large over all stages indicating that people need to make short trips, such as to the grocery store or to the healthcare, to maintain their daily lives. The sustained impact of Type 3 mobility indicates that the chances of people coming together during these activities to spread the virus remain high, especially during the epidemic phase of the virus. These findings suggest that the impact of different types of human mobility on the number of cases varies across pandemic stages, which provides a basis for decision-making on the use of NPIs when responding to a pandemic.

### 4.3 Type 1, 2, and 3 metrics vs. lockdown level

**Fig 5:**
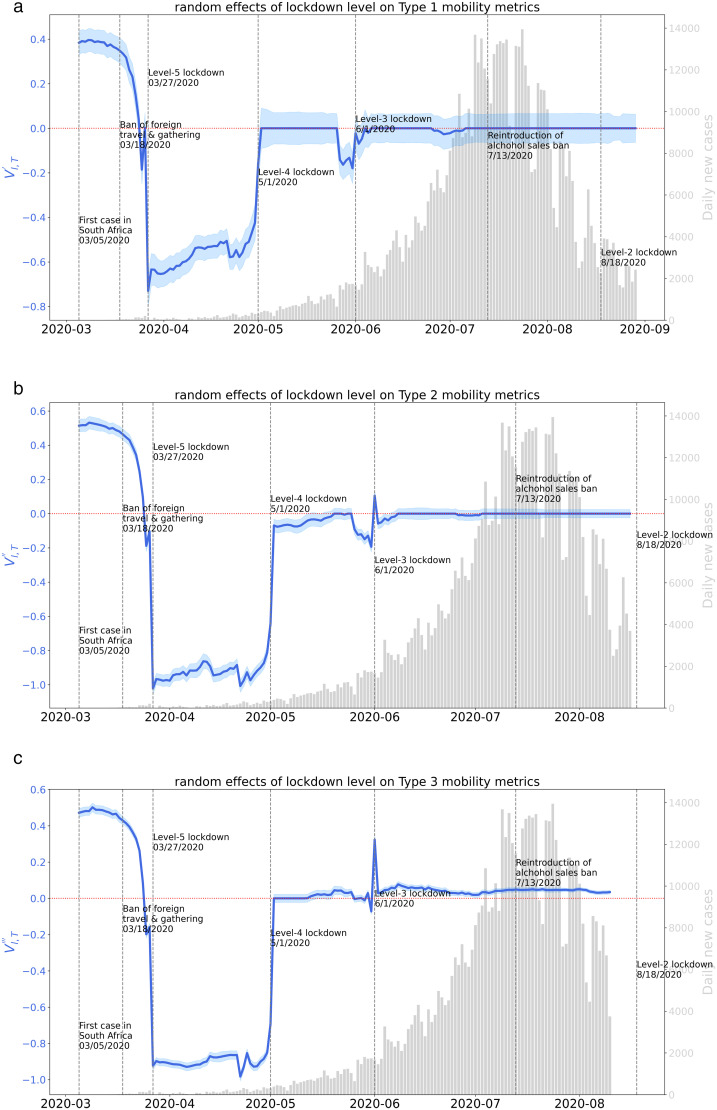
Random effects of lockdown level on natural-logged three types of mobility metrics.

Fig 5 visualizes the daily variation of dynamic coefficients in equations (2)–(4) in sub-figure (a), (b), and (c), respectively. The blue line represents the random intercept of lockdown level. The red dotted line is a horizontal line on value zero as reference. The grey bar in the background denotes the daily new COVID-19 cases of South Africa.

#### 4.3.1 The first lockdown.

The variation of random effects of lockdown level on Type 1, 2, or 3 mobility metrics are shown in Fig 5(a), (b), or (c). Over the period from March 27th to April 30th when the level 5 lockdown policy was in effect, the impact of the level 5 lockdown policy on all three types of trips was negative. It indicates that at the emergent stage of the pandemic, the government policy of South Africa played an important role in guiding people’s travel behavior. From the perspective of Type 1 metric, the negative effect of level 5 lockdown on Type 1 mobility metric (logarithmic) slowly rose from −0.72 to −0.42 during its in-effect period. It indicates that the lockdown policy’s impact on inter-provincial population flow gradually decayed as time went by. Negative effects can also be observed on Type 2 and Type 3 mobility metrics (logarithmic) with mean of −0.93 and −0.91, respectively.

#### 4.3.2 Social-distancing fatigue.

On May 1^st^, the lockdown level was lowered from level 5 to level 4. The effects of the level 4 lockdown policy on Type 1, Type 2, and Type 3 increased sharply to a value close to 0, −0.07, and 0, respectively on the following day. It indicates that the impact of the level 4 lockdown policy on people’s travel behavior became extremely limited, implying that the acceptance of the travel restriction policy was substantially decreased among the people. This surge can be explained as the reaction of people’s fatigue to social distancing as people have been restricted at home for a long time. Similar results can be found in other countries like the U.S. [21,53] and Switzerland [54]. From May 1^st^ to September 1^st^, as covered by the dynamic panel dataset, the impact of travel restriction policies on human mobility became minimal.

## 5. Conclusions

This pioneering study classifies human mobilities into three categories according to their origins and destinations (O-D) and analyzes their impact on viral transmission of SARS-CoV-2. This research demonstrates the application of structural equation modeling methodology on dynamic panel datasets to capture the dynamic impact of human mobility on the spreading of the SARS-CoV-2 virus, as well as the effect of restriction policies on human mobility in South Africa. The dynamic impacts have been analyzed and quantified along with multiple covariates, including sociodemographic, socioeconomics, date periodicity, and confounding factors such as lag effects and temporal autocorrelation. Results for most time windows are deemed statistically significant, accurate, and robust, offering valuable insights about human mobility patterns that can be directly utilized by epidemiologists to cross-validate or enhance the compartment models. For example, an SIR or SEIR (Susceptible-Exposed-Infectious-Recovered) [55,56] model divides a population into Susceptible, Exposed, Infectious, and Recovered groups to simulate the spread of infectious diseases, incorporating a latent period for exposure before individuals become infectious. In this model, the mobility data can provide more fine-grained information for researchers to discover how people make contact with each other, informing the model parameters and thus explaining the spatial and temporal patterns of outbreaks.

The main contributions can be summarized as below:

- By leveraging Location-Based Services (LBS) data, this study is the first to design three novel metrics that capture the spatial and temporal pattern of human mobility from various perspectives in Africa. These metrics offer a comprehensive view of mobility trends across regions of different administration levels, providing valuable insights for provincial-level and district-level governments to understand how travel behaviors vary across time and space. This approach lays the groundwork for further research on mobility-driven epidemic modeling and socio-economic analyses, highlighting the importance of localized mobility data in understanding public health and social dynamics in African contexts.
- To the best of our knowledge, this study is the first to quantify the dynamic effects of various types of human mobility on SARS-CoV-2 transmission in Africa. Specifically, we utilized Origin-Destination (O-D) data to create the three metrics, offering critical insights into interactions across various spatial regions, which would not be possible without such data, enabling us to capture cross-regional mobility dynamics that are essential for accurate modeling of transmission pathways.
- In modeling the epidemic, our approach also offers insights into how lockdown policies dynamically impact the three types of human mobility. The model reveals a phenomenon of social distancing fatigue, where adherence to lockdown measures decreases over time. These findings are consistent with previous research and dynamically quantify the impact of the lockdown policy on changes in travel behavior during prolonged lockdowns.
- This research provides new insights into epidemic modeling by integrating human mobility data to model viral transmission trends. The fusion of this data offers valuable information that can potentially be used to enhance traditional epidemic models, such as the SIR and SEIR models, in future applications.

The most highlighted findings in this research are about the dynamic relationship between the three types of mobility metrics and the daily new COVID-19 cases. Firstly, alternating and overlapping impacts were observed from the three mobility metrics on daily new cases during the stages of the epidemic. During the emergent stage, Type 1 and Type 3 metrics are associated with the daily new cases over longer periods compared to Type 2 mobility. Secondly, our model indicates that the Type 3 mobility metric had the longest and statistically significant influence on the daily new cases with a surge in the spreading stage followed by a gradual decay during the epidemic and controlled stages. This trend suggests that people gradually adapted to social distancing and other NPIs, leading to a reduction in the dynamic coefficient of Type 3 metric on daily new COVID-19 cases.

The results of this study have important implications for policy development in managing epidemic outbreaks by controlling human mobility. Consistent with previous studies [2,3], our analysis underscores the effectiveness of restricting human mobility to curb viral transmission, particularly during the emergent and spreading stages of epidemics. Notably, the long-term impact of the Type 3 mobility on new daily cases suggests that short-distance local trips driven by daily needs may continue to accelerate viral transmission even under restrictive conditions, suggesting that more stringent or more nuanced NPIs may be needed in such situations. In contrast, the alternating effects of the three types of mobility reveal different dynamics: for example, the Type 1 mobility characterized by inter-provincial travel has the earliest impact on new daily cases, implying that earlier restriction of inter-provincial travel significantly reduces the rate of virus transmission. In addition, our model highlights social distancing fatigue, which is particularly pronounced when the lockdown level is relaxed, during which it is difficult for policies to have an impact on mobility behavior. This emphasizes the need for proactive government measures, such as educating the public about the importance of NPI in advance and reinforcing public awareness through targeted campaigns, which can lead to long-term compliance. By utilizing these insights into mobility, South African policymakers can design more nuanced and timely interventions that effectively balance public health and societal needs.

Several limitations of this study are acknowledged and merit further exploration in future research. First, incorporating additional information related to trip origin-destination (O-D) patterns could enhance the understanding of the detailed effects of human mobility on daily new COVID-19 cases. For example, human mobilities within or between regions of high population density could be more impactful on virus transmission. Staying in specific locations may lead to higher rates of infection, such as tracking people who have spent time in health-care facilities. Analyzing previous trajectories could help identify specific types of locations that contributed to increased spatial-temporal contacts and facilitated the spread of the virus. Besides, this study aggregates and models the mobility data at the district level, which limits the ability to distinguish the effects of non-pharmaceutical interventions (NPIs), such as lockdown policy, on mobility from individual-led behavioral responses. Investigating individual-level behavioral responses is a critical area for future research, as it could provide deeper insights into how personal decisions interact with policy measures to shape mobility patterns and, consequently, virus transmission. Second, the penetration rate of mobile devices may be lower among younger, elderly, and lower-income populations, potentially introducing sample bias. This limitation could affect the representativeness of the mobility data and warrants further investigation to ensure equitable data coverage. Third, the COVID-19 case data in South Africa is currently available only at the provincial level. Access to finer-scale case data, such as at the district or municipal level, would enable a more granular analysis of the relationship between mobility and virus transmission, strengthening the study’s findings.

## Supporting information

S1 Fig P-values of the dynamic coefficients between daily new COVID-19 cases per 1 million population and Type 1 mobility metrics. (PNG)

S2 Fig P-values of the dynamic coefficients between daily new COVID-19 cases per 1 million population and Type 2 mobility metrics. (PNG)

S3 Fig P-values of the dynamic coefficients between daily new COVID-19 cases per 1 million population and Type 3 mobility metrics. (PNG)

S1 Table The VIF values of all variables of Daily New Cases sub-model in the SEM model. (DOCX)

S2 Table The VIF values of all variables of Type 1 Metric sub-model in the SEM model. (DOCX)

S3 Table The VIF values of all variables of Type 2 Metric sub-model in the SEM model. (DOCX)

S4 Table The VIF values of all variables of Type 3 Metric sub-model in the SEM model. (DOCX)
