# Supplementary figures and images for: Variant patterns and influence of inter-regional travel during the SARS-CoV-2 expansion in South Africa
Source: PLoS One. 2025 Nov 6;20(11):e0329621. doi: 10.1371/journal.pone.0329621 (PMC12591497; doi:10.1371/journal.pone.0329621)

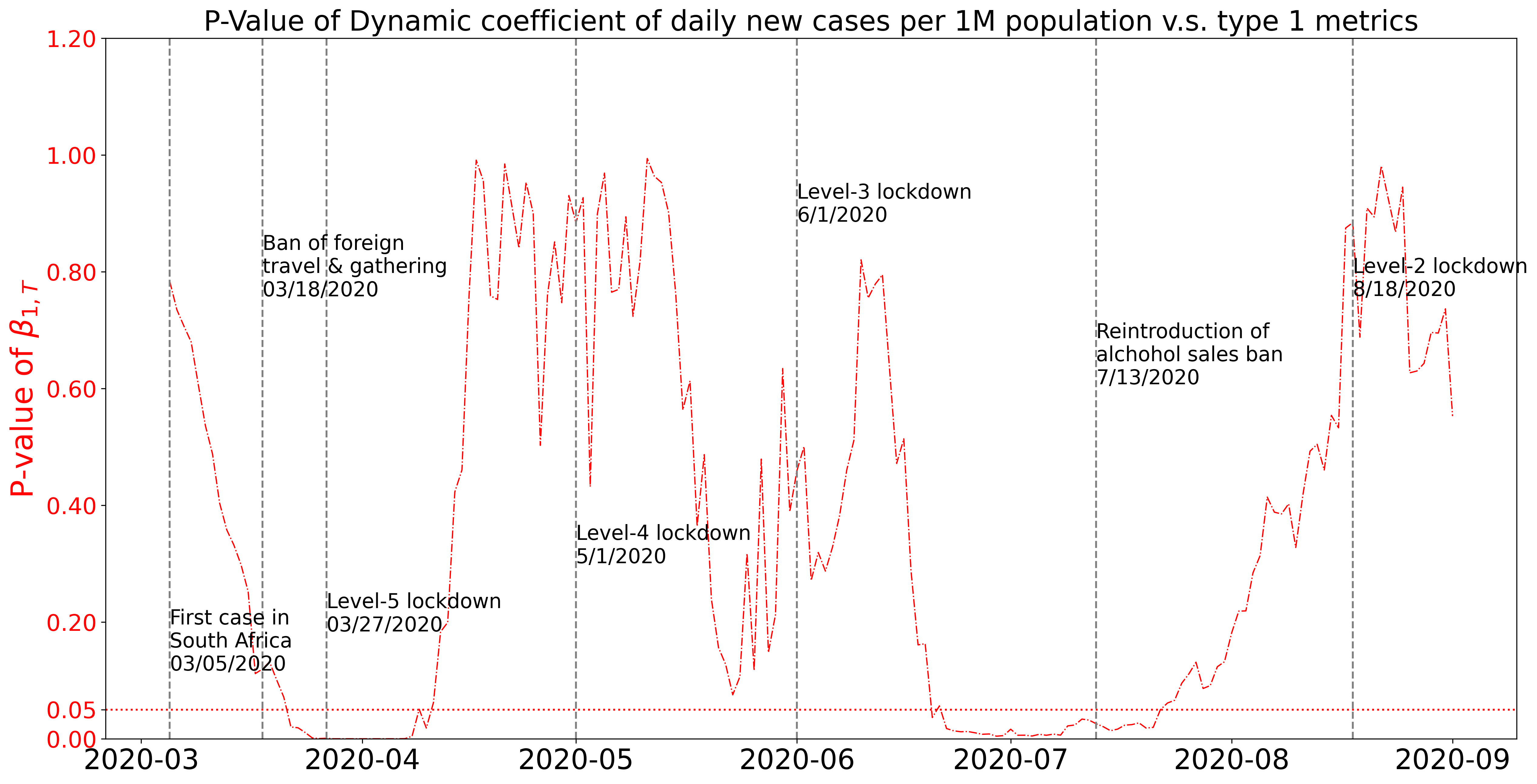

Supplement: S1 Fig — (PNG) [file pone.0329621.s001.png]

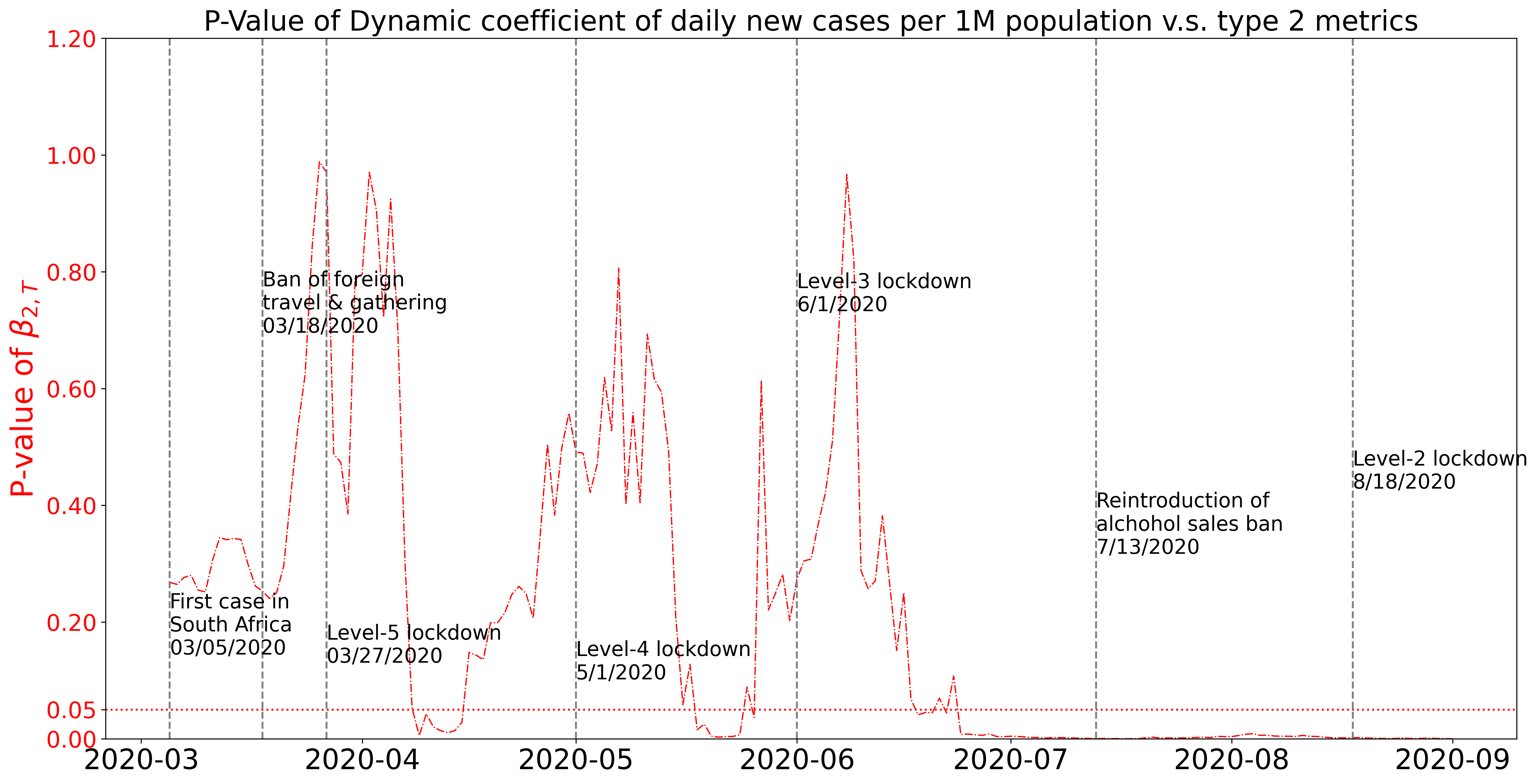

Supplement: S2 Fig — (PNG) [file pone.0329621.s002.png]

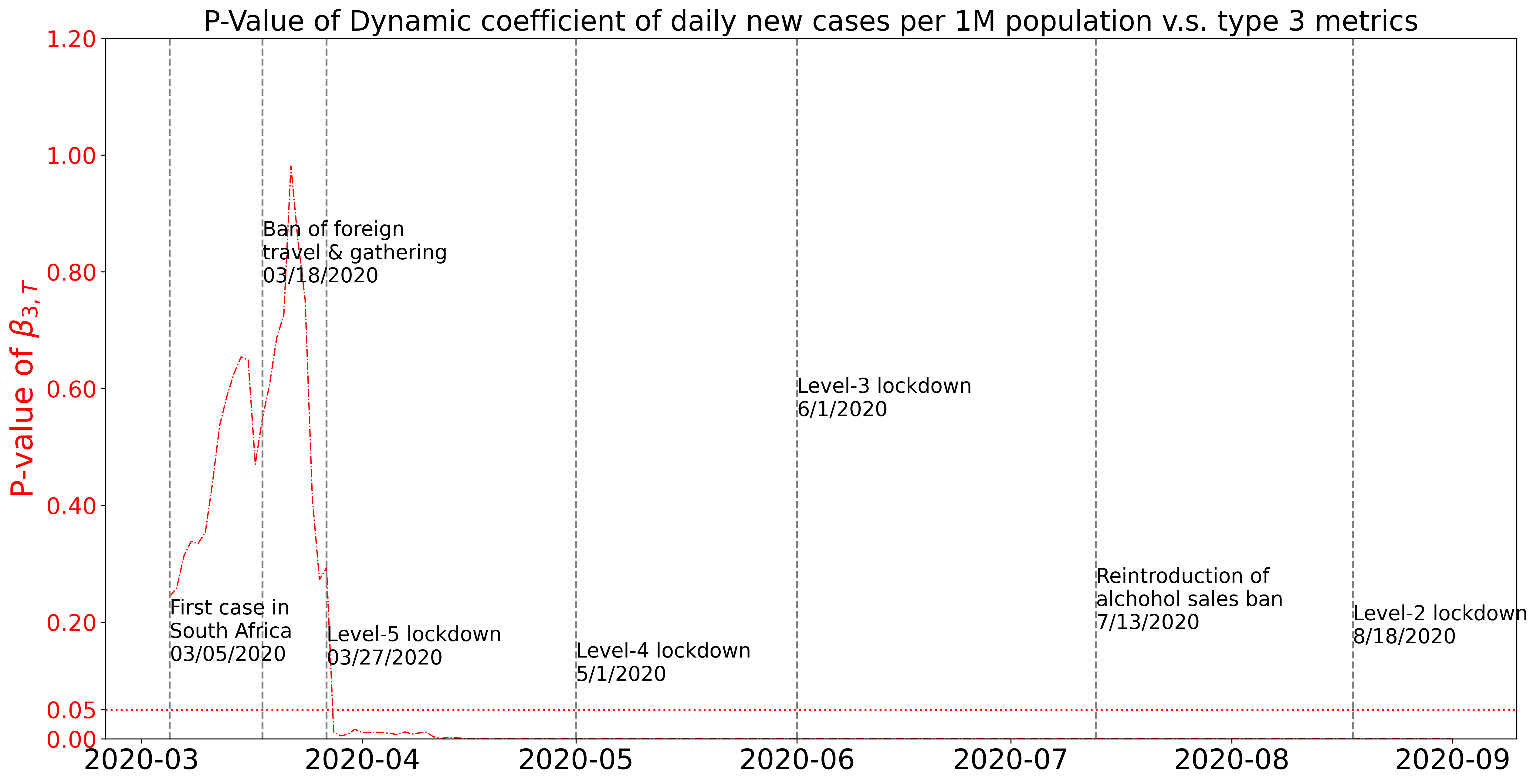

Supplement: S3 Fig — (PNG) [file pone.0329621.s003.png]
