# Supplementary material for: Variant patterns and influence of inter-regional travel during the SARS-CoV-2 expansion in South Africa
Source: PLoS One. 2025 Nov 6;20(11):e0329621. doi: 10.1371/journal.pone.0329621 (PMC12591497; doi:10.1371/journal.pone.0329621)
Supplement: S1 Table — (DOCX) [file pone.0329621.s004.docx]

Table S 1. The VIF values of all variables of Daily New Cases sub-model in the SEM model

| **Endogenous Variable** | **-** |
| --- | --- |
| Daily new cases | - |
| **Exogenous Variable** | **VIF** |
| Type 1 metric (3-day lag) | 1.57 |
| Type 2 metric (16-day lag) | 3.79 |
| Type 3 metric (22-day lag) | 2.52 |
| Daily new cases (1-day lag) | 1.07 |
| Weekend dummy | 1.01 |
| Relative Wealth Index | 2.29 |
| Percentage of population above age 65 | 1.46 |
| Percentage of male population | 1.75 |
