# Supplementary material for: Variant patterns and influence of inter-regional travel during the SARS-CoV-2 expansion in South Africa
Source: PLoS One. 2025 Nov 6;20(11):e0329621. doi: 10.1371/journal.pone.0329621 (PMC12591497; doi:10.1371/journal.pone.0329621)
Supplement: S3 Table — (DOCX) [file pone.0329621.s006.docx]

Table S 3. The VIF values of all variables of Type 2 Metric sub-model in the SEM model

| **Endogenous Variable** | **-** |
| --- | --- |
| Type 2 Metric (16-day lag) | - |
| **Exogenous Variable** | **VIF** |
| Daily new cases (16-day lag, 7-day moving average) | 1.01 |
| Type 2 Metric (17-day lag) | 1.37 |
| Relative Wealth Index | 1.79 |
| Percentage of population above age 65 | 1.41 |
| Percentage of male population | 1.45 |
| Weekend dummy (16-day lag) | 1.01 |
